# The Brazilian Longitudinal Study of Aging (ELSI-Brazil): Objectives and Design

**DOI:** 10.1093/aje/kwx387

**Published:** 2018-01-31

**Authors:** M Fernanda Lima-Costa, Fabíola Bof de Andrade, Paulo Roberto Borges de Souza, Anita Liberalesso Neri, Yeda Aparecida de Oliveira Duarte, Erico Castro-Costa, Cesar de Oliveira

**Affiliations:** 1 Instituto de Pesquisas René Rachou, Fundação Oswaldo Cruz, Belo Horizonte, Brazil; 2 Instituto de Comunicação e Informação Científica e Tecnológica em Saúde; 3 Faculdade de Ciências Médicas, Universidade de Campinas, Campinas, Brazil; 4 Escola de Enfermagem, Universidade de São Paulo, São Paulo, Brazil; 5 Department of Epidemiology and Public Health, University College London, London, United Kingdom

**Keywords:** aging, Brazil, chronic diseases, cohort study, epidemiologic methods, functioning, middle-income country

## Abstract

Brazil is experiencing among the world’s fastest demographic aging worldwide. This demographic transition is occurring in a context of few resources and great social inequalities. The Brazilian Longitudinal Study of Aging (ELSI-Brazil) is a nationally representative study of 9,412 people aged 50 years or older, residing in 70 municipalities across the 5 Brazilian regions. ELSI-Brazil allows investigations of the aging process, its health, psychosocial and economic determinants, and societal consequences. The baseline examination (2015–2016) included detailed household and individual interviews and physical measurements (blood pressure, anthropometry, grip strength, and timed walk and balance tests). Blood tests and sample storage were performed in a subsample of study participants. Subsequent waves are planned for every 3 years. The study adopts a conceptual framework common to other large-scale longitudinal studies of aging in the world, such as the Health and Retirement Study, allowing cross-national comparisons. The goal of ELSI-Brazil is not only to build an understanding of aging in a large, Western, middle-income country in a rapid demographic transition but also to provide scientific data to support and study policy changes that may affect older adults. We describe the methodology of the study and some descriptive results of the baseline survey.

Brazil, the world’s fifth most populous nation, with more than 200 million inhabitants, is experiencing among the fastest demographic aging worldwide, a trend that will accelerate during the 21st century (1). The new challenges posed by a large older population are a major concern in Brazil and other low- and middle-income countries (2–4). Promoting active aging (5) and building economic and social institutions to ensure income security and adequate health care (6) are crucial issues. Large-scale longitudinal data resources have the potential to support and evaluate short and long-term policies that affect older adults in those countries.

The rapid aging of the population in low- and middle-income countries is occurring in a context of few resources and great social inequalities (7, 8). The health of older populations in those countries, particularly among the poorer, tends to be substantially worse (9, 10), and the epidemiologic transition is leading to an increased exposure to risk factors for noncommunicable diseases, mostly in poor people (2, 11). Moreover, older people in those countries are prone to variable degrees of a double burden of diseases, characterized by the concomitance of noncommunicable and infectious diseases that, in turn, disproportionally affect those in worse socioeconomic circumstances (12). Therefore, low- and middle-income countries face the great challenge of improving the health of successive cohorts of older people equitably, as life expectancy increases, in an unfavorable context (3).

Brazil has one of the world’s highest levels of socioeconomic inequality (Gini coefficient = 0.53 in 2013) (13). It ranks 75th in the world on the Human Development Index (14). Life expectancy reached 75.5 years in 2016 (15), and the fertility rate declined from 6.28 children per woman in 1960 to 1.90 in 2010 (16). The current population aged 50 years or older has experienced dramatic political and social changes during their lifetimes. Brazil has rapidly transitioned from a low-income, primarily rural country in the mid-1950s to one of the top 10 economies in the world, with 84% of the population living in urban areas (17). Socioeconomic disparities in older adults are seen across a range of health conditions and in access to and use of healthcare (9, 18, 19). Brazil has the Unified Health System (in Portuguese, Sistema Único de Saúde (SUS)), designed to provide comprehensive and universal care through decentralized management and provision of health services that are free of charge at the point of delivery (17). Alongside SUS, 26% of Brazilian citizens have private health plans that allow them to access the private health sector (17). The subfinancing of SUS is an important issue (17). Compared with England, another country that provides universal health care, the per-capita health expenditure in Brazil is 3.7 times lower (US $875.00 vs. US $3,222.00) and the density of physicians is 17.0 (compared with 27.4/1,000 in England) (10).

The Brazilian Longitudinal Study of Aging (ELSI-Brazil) is the first large-scale longitudinal study of older adults in Brazil. ELSI-Brazil was designed to provide a national data resource on the aging process and health, psychosocial and economic determinants, and societal consequences. The goal of the study is not only to build an understanding of aging in a large, Western, middle-income country in rapid demographic transition but also to provide scientific data to support and study policy changes that may affect older adults. Here we describe the objectives and main aspects of the study.

## METHODS

### Study design

ELSI-Brazil is a nationally representative, population-based cohort study of people aged 50 years or older. The baseline survey was conducted between 2015 and 2016.

### Sample

The sample was designed to be representative of the noninstitutionalized population within the eligible age range. Due to the lack of a centralized and reliable household registry, Brazil’s national household surveys use the Brazilian Institute of Geography and Statistics (in Portuguese, Instituto Brasileiro de Geografia e Estatística (IBGE)) geographic operational base (16) for stratification and selection of areas. To ensure that the sample represents the urban and rural areas of the small, medium, and large municipalities, the ELSI-Brazil sampling used a design with selection stages, combining stratification of primary sampling units (municipalities), census tracts, and households. The municipalities were allocated to 4 strata depending on their population size. To decide the size of the municipality and the number of municipalities allocated to each stratum, the Lavallée and Hidiroglou (20) method of stratum construction was performed using the R package stratification (R Foundation for Statistical Computing, Vienna, Austria). The 4 strata were categorized as follows: first stratum (≤26,700 inhabitants from 4,420 municipalities); second stratum (26,701–135,000 inhabitants from 951 municipalities); third stratum (135,001–750,000 inhabitants from 171 municipalities); and fourth stratum (>750,000 inhabitants from 23 municipalities). For the first 3 strata (municipalities up to 750,000 inhabitants), the sample was selected in 3 stages. In the first stage, 18 municipalities were selected in the first stratum, 15 in the second, and 14 in the third. In the second stage, 8 census tracts were selected in each municipality, and finally, the households were selected in each census tract. In the fourth stratum, which included the largest municipalities, the sample selection was done in 2 stages. In the first stage, 176 census tracts were selected, and in the second stage households were selected. All residents in the selected households aged 50 years or older were eligible for interview and other procedures, and a subsample was selected for blood collection (see below).

**Figure 1.**
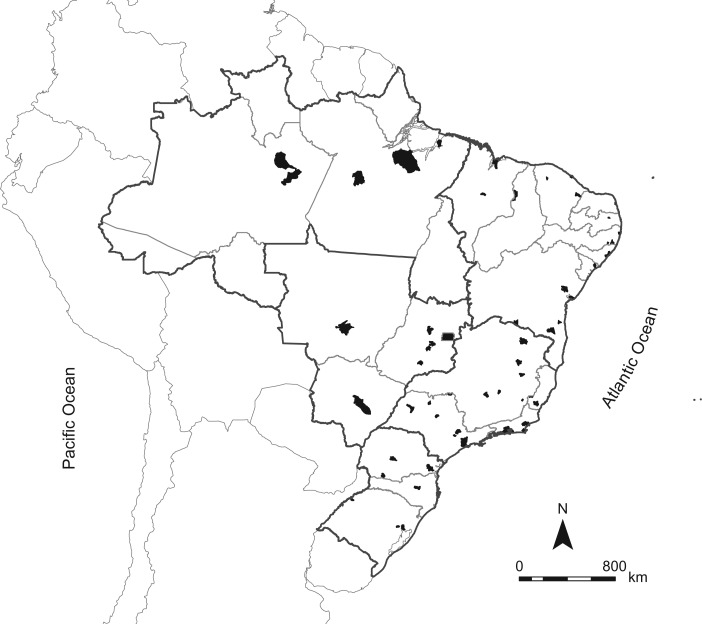
Map of Brazil showing municipalities participating in the Brazilian Longitudinal Study of Aging (ELSI-Brazil), 2015–2016.

We used an inverse sampling design to avoid an increase in the sample size to compensate for nonresponses (21, 22). The inverse sampling design enables investigators to define how many units need to be observed in order to obtain a prespecified number of successes, that is, interviews to be performed. Application of this method in ELSI-Brazil consisted of sequentially visiting the previously selected households until reaching the planned number of interviews. The planned number of interviews was 10,000 (9,412 participated), residing in 70 municipalities from different Brazilian regions. This sample size allows an estimated prevalence of 1% (sample error = 0.25%), or a prevalence of 5% (sample error = 0.55%), at a level of significance of 95% and an effect sample design of 1.5. For inequality comparisons, it will be possible to identify differences of 3.6% between the top and bottom quintiles, for a prevalence of 10% with a power test of 80%. Figure 1 illustrates the distribution of municipalities included in the survey.

### Weighting

Sample weights were derived to account for differential probability of selection and differential nonresponse. Correct use of weights is essential to population inference because the sample is not self-weighting by design. Because ELSI-Brazil has a complex sample design, analyses should account for geographical stratification and clustering in the estimation of standard errors. The mean natural and calibrated weights were: 3,501.6 (standard deviation (SD), 1,695.4) and 4,551.5 (SD, 2,515.9) for the first stratum; 3,568.8 (SD, 2,349.3) and 4,844.9 (SD, 3,464.1) for the second; 4,341.5 (SD, 3,040.1) and 5,706.3 (SD, 4,037.5) for the third; and 2,627.5 (SD, 1,497.5) and 3,573.0 (SD, 2,216.1) for the fourth.

### Baseline measurements

Baseline measurements included: 1) household interview; 2) individual interview; 3) physical measurements; 4) blood tests; and 5) samples storage for future analysis. In households with more than 1 resident, the household interview was done with the adult chosen by the other residents as the one who would best provide the required information. All residents aged 50 or older were eligible for individual interview and physical measurements, and a subsample of 4,500 persons (i.e., those residing in a random sample of 50% from the previously selected municipalities) was selected for blood collection. A proxy was required to answer the individual interview when needed, and this was annotated properly.

The household interview included measures of house characteristics and accessibility, as well as detailed information on housing assets, consumption, and income of each resident. Housing assets included house property; payment of mortgage; rental payment; house market value; other house, flat, land, or farm property and their market value; appliances in the household; vehicle property and market value; and domestic employees in the household. Consumption included money spent on eating out, utilities (electricity, water, cooking gas), condominium fees, fuel and maintenance or repair of vehicles, telephone bills, entertainment, property and vehicle tax bill, purchase of motor vehicles, education/school, private health plan, and other expenses. For those aged 50 years or older, individual income information included employment, private and state pension, and other benefits. For residents aged 17–49 years, income from all sources was considered. The measures obtained in the household interview are summarized in Web Table 1 (available at https://academic.oup.com/aje).

Individual interviews included a broad range of topics related to an individual’s demographic characteristics, neighborhood, discrimination, living and health conditions in early life (childhood questionnaire), work and retirement, family transfers, health behaviors, women’s health, physical and mental health, oral health, functioning and helpers, psychosocial measures, and use of medications and of health services. Health measures included the broad geriatric syndromes of frailty, continence, gait, balance and falls (23), and history of medical diagnosis for an array of illnesses. The interview also included information on treatment for major chronic conditions and the use of preventive services for hypertension, diabetes, dyslipidemia, breast cancer, and cervical cancer. The section on functioning included measures of mobility and the person’s ability to perform basic (BADL: bathing, dressing, feeding, etc.) and instrumental (IADL: shopping, managing money, etc.) activities of daily living. Furthermore, this section included information on the person’s ability to perform advanced activities of daily living (AADL: social activities, productive activities, and entertainment) (24) and detailed information on sources of help to perform basic activity tasks. The section on the use of health services comprised access/barriers, expenses, doctors’ visits, hospitalization, and perceived quality of care received. Measures of psychosocial factors included sociability, social support, subjective well-being, critical life events, religiosity, and quality of life in terms of control, autonomy, self-fulfillment, and pleasure (CASP-19). The 8-item Center for Epidemiologic Studies–Depression Scale (CES-D-8) was used to assess depressive symptoms (25, 26). Cognition assessments were based on questionnaires used in other large-scale longitudinal studies in the world (27–31), comprising memory (self-rated memory, orientation in time, word-list learning, prospective memory, names of people and things) and executive function (word-finding/verbal fluency). When a participant was not able to respond to the cognitive function module, a proxy was required to rate the current participant’s memory, and in relation to the last contact. The main exposure domains ascertained through the individual interview are shown in Web Table 2. The full household and individual questionnaires are available at the ELSI-Brazil homepage (http://elsi.cpqrr.fiocruz.br/).

A comprehensive set of physical measurements and blood tests was carried out. Interviewers administered physical tests at the respondent’s home. The anthropometric parameters included weight, height, and waist and hip circumferences, and were obtained in duplicate. Resting blood pressure was measured 3 times in the seated position after 5 minutes of rest; the average of the second and third measures must be used in the analysis. Other physical measures included grip strength, timed walk, and balance tests. A trained laboratory technician obtained nonfasting venous blood samples in a separate visit to the participant’s household. All biological specimens were sent to the central laboratory (see below). The following tests were performed: total cholesterol, high-density lipoprotein (HDL) cholesterol, low-density lipoprotein (LDL) cholesterol, urea, creatinine, ferritin, thyroid stimulating hormone (TSH), glycated hemoglobin, vitamin D, and hemogram. Serum and plasma aliquots are stored in liquid nitrogen at the Oswaldo Cruz Foundation, Belo Horizonte, Brazil. DNA extraction and genome assays on those aliquots are planned. Further details are described in the Web Table 3.

### Cohort surveillance and follow-up

Subsequent waves are planned for every 3 years. Household interview, individual interview, and physical measurements will be repeated at each wave. Sample refreshment is planned every 6 years, at which laboratory tests and aliquots storage shall be repeated. In addition, as an attempt to reduce attrition rates, the following precautions have been taken: 1) during the baseline interview, telephone numbers and addresses of the participant’s network (family, friends, and neighbors) were recorded; 2) between the interviews, telephone calls to monitor changes of addresses and vital status will be performed; 3) linkage to the National Mortality System will be performed. In order to track those participants who were not located through the above-mentioned procedures, a census tract visit (participant’s household and neighboring households if necessary) is planned for subsequent waves.

### Quality assurance and control

A series of small pilot studies and a further larger pilot study were performed to identify and correct potential problems in the data collection instruments and procedures. Central training and certification of interviewers were performed according to the study protocol. The standardized study procedures are detailed in the ELSI-Brazil operations manual (available at the ELSI-Brazil homepage: http://elsi.cpqrr.fiocruz.br/). Interviews were recorded, when authorized by the respondent, and a sample of the recordings was reviewed by trained supervisors. Baseline blood collection and laboratory assays were performed by a private contractor. This laboratory is accredited by the Ministry of Health (Brazil), and by the College of American Pathologists (United States) and the Clinical Laboratory Accreditation Program (United States) (available by request). The blood samples were collected at the participant’s home. After preparation, samples were shipped (by air, depending on the distance) to the laboratory situated in São Paulo, Brazil. Best practices were followed to ensure quality and viability of the samples, including packing in dry ice and temperature monitoring along the transportation route (the transportation protocol is available by request, in Portuguese).

### Study management

ELSI-Brazil is conducted by the Oswaldo Cruz Foundation (FIOCRUZ) and Federal University of Minas Gerais (UFMG), Belo Horizonte, Brazil. The study’s steering committee includes researchers from these institutions and other consultants in Brazil and other countries. This committee developed the operation manuals and was responsible for training and certifying staff. The research center has developed data entry and management systems, as well as a system for transmission of interviews in real time.

### Ethical approval

ELSI-Brazil was approved by the ethics board of FIOCRUZ, Minas Gerais (Certificado de Apresentação para Apreciação Ética: 34649814.3.0000.5091). Genotyping of the cohort population was approved by Brazil’s national research ethics committee (Certificado de Apresentação para Apreciação Ética: 63725117.9.0000.5091). Participants signed separate informed consent forms for the interviews, physical measurements, and the laboratory assays, authorized sample storages, and access to administrative records.

### Selected baseline characteristics of study participants and representativeness

We used data from the recent Brazilian National Health Survey (in Portuguese, Pesquisa Nacional de Saúde (PNS)), conducted in 2013 (30), to compare selected baseline characteristic of ELSI-Brazil’s participants at baseline with those of participants of that survey. All PNS data are publically available at http://www.ibge.gov.br/home/estatistica/populacao/pns/2013/.

As shown in Table 1, ELSI-Brazil’s participants were similar to those of PNS regarding age, sex, number of residents in the household, rural residence, residence across Brazilian regions, and level of education. Marital status differed between the 2 populations in that the proportion of married people was slightly higher in ELSI-Brazil.

**Table 1.** Selected Baseline Sociodemographic Characteristics of Participants in the Brazilian Longitudinal Study of Aging (2015–2016) Compared With Those Aged 50 Years or Older From the Brazilian National Health Survey (2013)

Characteristic	ELSI-Brazil (*n =* 9,412)^a^		PNS (*n =* 45,154)^a^	
	Mean or %^b^	95% CI	Mean or %^b^	95% CI
Mean age, years	62.9	62.1, 63.8	62.6	62.4, 62.8
Female sex	54.0	51.0, 56.9	55.0	54.5, 55.5
Mean no. of people in the household	3.1	3.1, 3.2	3.1	3.0, 3.1
Marital status				
Married	63.5	60.5, 66.3	56.7	55.7, 57.5
Windowed	14.7	12.9, 16.8	17.6	17.0, 18.2
Single/divorced	21.8	20.0, 23.7	25.9	25.2, 26.6
Rural residence	15.3	11.2, 20.6	14.1	13.5, 14.8
Geographic region				
North	5.6	2.3, 12.8	5.6	5.3, 5.9
Northeast	24.1	15.9, 34.8	24.6	23.9, 25.3
Center-West	6.6	3.0, 13.8	6.7	6.5, 7.1
Southeast	47.2	35.6, 59.1	47.5	46.5, 48.5
South	16.6	8.8, 29.1	15.6	14.9, 16.3
Schooling, years				
<8	64.6	61.3, 67.4	61.9	60.7, 63.1
8–10	11.9	10.6, 13.3	10.5	10.0, 11.2
≥11	23.8	21.7, 26.0	27.6	26.5, 28.7

Abbreviations: CI, confidence interval; ELSI-Brazil, Brazilian Longitudinal Study of Aging; PNS, Brazilian National Health Survey.

^a^ Unweighted.

^b^ All results are percentages, except when specified. All estimates considered the complex sample design and survey weights.

As shown in Table 2, ELSI-Brazil’s participants were similar to those of PNS regarding prevalence of self-reported diabetes, stroke, asthma, cancer, current smoking, alcohol consumption, mean systolic blood pressure, diastolic blood pressure, body mass index, and waist circumference. The prevalence of self-reported arthritis was higher in ELSI-Brazil than in PNS. The prevalence of hypertension was slightly higher in ELSI-Brazil.

**Table 2.** Selected Health Conditions and Lifestyle of Participants in the Brazilian Longitudinal Study of Aging (2015–2016) Compared With Those Aged 50 Years or Older From the Brazilian National Health Survey (2013)

Characteristic	ELSI-Brazil (*n* = 9,412)^a^		PNS (*n* = 20,207)^a,b^	
	Mean or %^c^	95% CI	Mean or %^c^	95% CI
Prior medical diagnosis				
Diabetes	15.8	14.6, 17.1	15.3	14.4, 16.2
Stroke	5.3	4.7, 6.0	3.7	3.2, 4.1
Asthma	4.9	4.2, 5.7	4.1	3.7, 4.6
Cancer	5.3	4.6, 6.0	4.2	3.7, 4.7
Arthritis	21.0	19.4, 22.7	13.6	12.8, 14.4
Lifestyle				
Current smoking	15.8	14.4, 17.3	15.3	14.4, 16.2
Consuming alcohol at least once a month	23.1	20.6, 25.8	20.0	18.6, 20.8
Blood pressure and anthropometric measurements				
Mean SBP in mm Hg	135.8	134.8, 136.9	134.7	134.2, 135.3
Mean DBP in mm Hg	78.6	78.0, 79.3	81.1	80.8, 81.2
Hypertension^d^	63.2	60.4, 64.2	58.3	57.1, 59.4
Mean body mass index^e^	27.8	27.6, 28.0	27.2	27.1, 27.3
Mean waist circumference in centimeters	93.5	92.9, 94.1	95.5	95.2, 95.9

Abbreviations: CI, confidence interval; DBP, diastolic blood pressure; ELSI-Brazil, Brazilian Longitudinal Study of Aging; PNS, Brazilian National Health Survey; SBP, systolic blood pressure.

^a^ Unweighted.

^b^ Subsample of study participants.

^c^ All results are percentages, except when specified. All estimates considered the complex sample design and survey weights.

^d^ Defined as SBP ≥140 mm Hg and/or DBP ≥90 mm Hg and/or treatment.

^e^ Calculated as weight (kg)/height (m)^2^.

Reported limitations in performing most activities of daily living were similar between the 2 populations (e.g., eating, bathing, using the toilet, walking across a room, shopping, managing money, and using transportation). Limitation in dressing was higher in ELSI-Brazil compared with PNS (Table 3).

**Table 3.** Prevalence of Limitations in Selected Activities of Daily Living^a^ of Participants in the Brazilian Longitudinal Study of Aging (2015–2016) Compared With Those Aged 60 Years or Older^b^ From the Brazilian National Health Survey (2013)

Characteristic	ELSI-Brazil (*n* = 5,375)^c^		PNS (*n* = 9,920)^c^	
	%^d^	95% CI	%^d^	95% CI
Eating	2.9	2.4, 3.5	5.0	4.6, 5.4
Bathing	7.3	6.5, 8.2	7.5	7.0, 8.1
Using the toilet	4.8	4.2, 5.6	6.6	6.1, 7.1
Dressing	14.6	13.2, 16.2	9.2	8.6, 9.8
Getting in and out of bed	10.7	9.6, 11.8	8.8	8.2, 9.4
Walking across a room	7.3	6.5, 8.3	8.8	8.2, 9.4
Shopping	19.7	17.9, 21.5	17.9	17.1, 18.7
Managing money	13.5	11.8, 15.4	12.8	12.1, 13.5
Using transportation	26.9	24.8, 29.1	22.0	20.6, 25.0

Abbreviations: CI, confidence interval; ELSI-Brazil, Brazilian Longitudinal Study of Aging; PNS, Brazilian National Health Survey.

^a^ Any difficulty in performing the task.

^b^ All participants aged 60 years or older from both surveys; PNS has information on these variables only from older participants.

^c^ Unweighted.

^d^ All estimates considered the complex sample design and survey weights.

There was a graded association between limitations in activities of daily living tasks and educational level (Table 4). For all tasks, the prevalence of limitations decreased from those with less than 8 years of education, to those with 8–10 years, and then to those with ≥11 years of education. These results are in agreement with previous publications, showing an inverse association between limitations in activities of daily living and household income and/or education among older Brazilians (10, 18, 19).

**Table 4.** Prevalence of Limitations in Selected Activities of Daily Living^a^, According to Years of Education, of Participants Aged 50 Years or Older in the Brazilian Longitudinal Study of Aging, 2015–2016

Limitation	Schooling, years					
	<8 (*n* = 6,308)^b^		8–10 (*n* = 1,027)^b^		≥11 (*n* = 2,015)^b^	
	%^c^	95% CI	%^c^	95% CI	%^c^	95% CI
Eating	2.9	2.3, 2.5	2.3	1.4, 3.7	0.7	0.04, 1.2
Bathing	7.5	6.8, 8.4	4.7	3.4, 6.3	2.6	2.0, 3.7
Using the toilet	5.4	4.7, 6.1	1.9	1.2, 3.1	1.5	1.0, 2.2
Dressing	15.3	14.1, 16.5	10.0	7.8, 12.7	6.8	5.7, 8.3
Getting in and out of bed	11.1	10.0, 12.2	6.0	4.5, 8.1	3.8	3.0, 4.8
Walking across a room	7.1	6.3, 8.0	4.1	2.7, 6.2	1.9	1.4, 2.5
Shopping	20.3	18.6, 22.1	11.6	9.1, 14.7	5.5	4.5, 6.7
Managing money	13.8	12.4, 15.6	6.2	4.3, 8.9	3.9	2.9, 5.3
Using transportation	26.1	23.9, 28.4	15.6	12.6, 19.1	11.1	9.4, 13.0

Abbreviations: CI, confidence interval.

^a^ Any difficulty in performing the task.

^b^ Unweighted.

^c^ All estimates considered the complex sample design and survey weights. All differences according to education were statistically significant: *P* < 0.001.

## DISCUSSION

ELSI-Brazil provides nationally representative data that enable inferences to be made about the Brazilian population aged 50 years or older. The study adopts a conceptual framework and approach common to other large-scale longitudinal studies of aging in the world, such as the Health and Retirement Study (27), the English Longitudinal Study of Ageing (28), the Mexican Health and Aging Study (29), and the China Health and Retirement Longitudinal Study (30), among others (31), allowing cross-national comparisons of the findings from ELSI-Brazil with those from other studies. In addition, ELSI-Brazil is part of the USC Global Gateway project, which provides harmonization tools for working with multiple country data sets (https://g2aging.org/). The research also includes new topics to capture Brazil-specific interests.

Recent economic crises and proposed reforms in social security may have unknown implications for the welfare of older Brazilians. ELSI-Brazil represents a unique opportunity to investigate the impact of such reforms. The topics covered include an array of successful aging measures and their determinants (e.g., economic, physical and social environment, familial, health behaviors, physical health, psychosocial, physical and cognitive functioning, use of health services and social programs), and events and transitions (e.g., disability, retirement, widowhood, and institutionalization). The study includes a wide range of socioeconomic measures and adopts new techniques for assessing income and wealth that have been previously shown to improve data quality (27–31). Changes in the labor market, retirement policies, and pensions are expected with population aging (4). The study is uniquely positioned to provide insights into the causes and consequences of retirement as the population ages in Brazil.

Disability is a major concern in the context of population aging (32). The extent of an individual’s disability is an important determinant of whether or not they require long-term care. Therefore, the increasing number of older persons generates concern in part because of the related increase in demand for and cost of long-term care (33–35). Long-term care at a household level in Brazil is fragmented, and there is no national policy to support such care (19, 36). A recent study estimated that about 6.5 million Brazilians aged 60 years or older need help to carry out activities of daily living, 360,000 did not get help although they needed it, and at least 5.7 million relatives or friends provide informal (nonpaid) care for older adults (19). These figures give some dimensions of the challenge facing Brazilian society to guarantee long-term care at the household level for older people in a context of rapid aging, reduction of the size of families (and thus, of the availability of informal caregivers), and economic crisis. ELSI-Brazil is designed to provide comprehensive information on activity-of-daily-living disability and sources for long-term care at the household level. Following the framework of World Health Organization’s International Classification of Functioning, Disability, and Health (ICF) (37), the study also includes information on functioning in a set of domains that range from hearing, seeing, and moving around to cognition and affect. A topic of growing interest is cognitive function, and ELSI-Brazil has cognitive measures obtained by using an internationally validated instrument (38). Well-being in old age is an endpoint and a right (4). The study includes an array of psychosocial measures that allows examination of both the associations between several dimensions of well-being and health and the determinants of those events. Chronic conditions and diseases are measured not only by self-report but also objectively. Given the exploratory nature of the study, the possibilities are extensive.

ELSI-Brazil is funded by the Brazilian Ministry of Health, and naturally, there is a great interest in information that can be useful for health planning and evaluation. The Madrid International Plan of Action on Aging called for the elimination of social and economic inequalities in access to health care and the development of health and long-term care to meet the needs of older people (39). To meet these needs, health systems must overcome challenges faced by older persons, including limited financial resources, discrimination, multimorbidity, limited mobility, and frailty (3). A major challenge is the provision of age-appropriate primary care (3). As part of the national public health system (SUS), Brazil rolled out the Family Health Program (FHP) in 1994 as a new model of community-based primary health care. In addition to regular home-based visits, all services and some medications are provided free of charge. Currently, FHP is implemented in nearly every municipality and reaches over 100 million people (40). Despite this rapid expansion, especially in primary health care, concerns remain about the Brazilian health system’s ability to improve equity in access and to provide high-quality care for chronic conditions (17, 41–43). ELSI-Brazil includes a broad range of indicators of access and use of health services and programs as well as indicators of the quality of primary care provision (44), user satisfaction with the care they receive, and health-care expenditures.

Brazil offers a rare opportunity to explore ethnoracial disparities on health and related outcomes in admixed populations. The Brazilian population originated from African, European, and Native American ancestral roots (45). The absence of legal segregation and other factors contributed to an emergence of a population with variable degree of intermixing (46). Brazilians self-reporting as black and brown are more likely to have lower income and education (47–50), to report experiencing discrimination (50, 51), and have more negative health-related outcomes (50, 52, 53). A strength of ELSI-Brazil is the use of genomic measures of ancestry (coming soon) and not only self-classification, which is prone to misclassification, particularly in admixed populations (46). Therefore, the study has the potential to contribute to a better understanding of the complex relationships between ethnoracial background and health conditions and their determinants in later life.

The dengue virus was reintroduced into Brazil in 1986, and by 1995 it had spread throughout the country (54). Chikungunya virus was introduced by 2013 at unprecedented speed and scale (55). A major epidemic of neonates with microcephaly linked to Zika virus was detected soon after, in 2015, and declared by the World Health Organization as a Public Health Emergency of International Concern (56). Chikungunya is characterized by a sudden onset of fever, accompanied by moderate to severe joint pain. Those symptoms may be confused with those of dengue in areas were both viruses circulate, but in contrast with dengue, the joint pain can last much longer, up to several years, and can be highly debilitating (55). In addition to microcephaly in neonates, Zika virus has been found to be associated with Guillain-Barré syndrome (apparently with very low incidence) (57), and experimental evidence suggests that the virus could attack cells in the hippocampus in adult mice, which raises questions about how the virus might affect human adults (58). ELSI-Brazil allows for the identification of cohort participants with dengue, chikungunya, or Zika by linkage with the national surveillance health system and by serological array in storage aliquots (planned). Therefore, ELSI-Brazil has the potential to explore possible long-term consequences of those illnesses in older adults.

In summary, ELSI-Brazil allows innovative investigations of the aging process, its determinants, and societal consequences. The study is part of an international effort (28–31) with the potential to provide new information that will be useful for planning and evaluation of social and health policies in Brazil and other low- and middle-income countries.

## Supplementary Material

Web Material

## Abbreviations

ELSI-Brazil: Brazilian Longitudinal Study of Aging
PNS: National Health Survey
SD: standard deviation
SUS: Unified Health System
