# Supplementary material for: The Brazilian Longitudinal Study of Aging (ELSI-Brazil): Objectives and Design
Source: Am J Epidemiol. 2018 Jan 31;187(7):1345–53. doi: 10.1093/aje/kwx387 (PMC6031009; doi:10.1093/aje/kwx387)
Supplement: Web Material [file kwx387lima-costawebmaterialfinal.pdf]

**WEB MATERIAL**

Web Table 1 - Summary of ELSI-Brazil baseline household interview by questionnaire module

| | |
|---|---|
| Enrollment section | Number of people in the household, their names, data of birth and relationship with the head of family |
| Section A: Household characteristics | Access to the household (staircase and number of steps), predominant material on the outside walls; street (asphalted or paved); number of rooms (total, bathrooms, bedrooms); source of water; adaptations for people with limited mobility (type by room); registration with the Family Health Program and visits from a Community Health Agent |
| Section B: Assets | House property; payment of mortgage; rental payment; house market value; other house, flat, land or farm property and their market value; appliances in the household (refrigerator, washing machine, microwave and others); vehicles property and their market value; domestic employees in the household (number and salary). |
| Section C: Household expenses (consumption) | Number of people had regular meals in the household; consumption of food produced by a household member and their market values; spent on eating out; spent with electricity/water/cooking gas, condo fees, fuel and maintenance or repair of vehicles, telephone bills, entertainment, property tax bill (IPTU), automotive ownership tax (IPVA), purchase of motor vehicle, education/school; affiliation with private health plan and fees; other expenses. |
| Section D: Income | Residents aged 50 years and older: income from salaries or self-employment; retirement and other benefits; alimony/monetary donation, rental/leases, savings accounts/other financial investments, other sources. Residents aged 17-49 years: income from all sources. Total household income. |

Web Table 2 - Summary of ELSI-Brazil baseline individual interview (residents aged 50 years and older) by questionnaire module

| | |
|---|---|
| Sections E: Demographics and background | Nativity and immigration; citizenship; state of birth; current and past marital status; ethno-racial background; formal and informal education; number of children, grandchildren and siblings; father's and mother's education and background. |
| Section F: Neighborhood | Perception of neighborhood; crime and violence. |
| Section G: Discrimination | Perception of discrimination. |
| Section H: Mini childhood life history | Family structure and economic conditions of family at the age of 10; family's socioeconomic status and respondent's health conditions up to the age of 15. |
| Section I-J: Work and retirement | Employment status and history; social security and/or private pension plans; retirement plans. |
| Section K: Family transfers | Transfers to and from children/grandchildren/great grandchild of money. |
| Section L: Health behaviors | Physical activities; food consumption; alcohol consumption and smoking. |
| Section M: Women's health | Menarche and menopause; reproductive history; hysterectomy; preventive examinations (Pap smear and mammogram). |
| Section N: General health and diseases | General health (self-rated health, number of days with good physical and mental health); vision and hearing; falls and joint surgeries; major chronic diseases (previous medical diagnosis, preventive services and treatment of hypertension, diabetes, and cholesterol); other chronic diseases (previous medical diagnosis), family's history of cardiovascular diseases and diabetes; history of bypass surgery/stent/angioplasty; flu shot; phenotype of frailty; sleep. |
| Section O: Oral health | Self-reported clinical measures (number of teeth, use of dental prostheses, need for dental treatment); self-rated oral health, use of dental services and impact of oral health on quality of life. |
| Section P: Functional limitations | Mobility; instrumental activities of daily living; basic activities of daily living; advanced activities of daily living; |

| | |
|---|---|
| | helpers. |
| Section Q and QP: Cognition | Memory (self-rated, orientation in time, word-list learning: verbal learning and recall - immediate and delayed), prospective memory; names of some people and things); executive function (word-finding: verbal fluency – animal naming). Proxy rating of global memory (present and past) and respondent's change in memory. |
| Section R: Depressive symptoms | The eight-item Center for Epidemiologic Studies Depression scale (CES-D8) |
| Section S: Psychosocial | Sociability (social relationships), availability and quality of social support within networks of informal relationships; subjective well-being indicated by life satisfaction; critical life events in the last 12 months; religiosity (religious affiliation, frequency of religious activities and meanings of religiosity); quality of life in terms of control, autonomy, self-fulfillment and pleasure (CASP-19). |
| Section T: Use of medications | Current number of medications of regular use; expenditure with medications; barriers to get medications. |
| Section U: Use of health services and health expenses | Affiliation to private health plans; doctor visits; regular source of care (perception of quality and barriers); hospitalizations; emergency care; health expenses. |

Web Table 3 - Summary of ELSI-Brazil baseline physical examinations and performance, and blood assays (residents aged 50 years and older)

| Procedure | Description (equipment) |
|---|---|
| Physical examinations and performance [a] | |
| Anthropometry | Weight, height, and measurement of waist and hip circumference: 2 measures each (Scale: Seca 813, Germany; Stadiometer: Nutri Vita, Brazil) |
| Blood pressure (systolic and diastolic) | 5 minutes' rest, 3 determinations (Oscilometric sphygmomanometer: Omron HEM 7200, Japan) |
| Grip strength | Stronger arm with elbow about 90% (Dynamometer: Saehan Sh5002, Belgium) |
| Balance test | Feet side by side, one foot ahead of the other, and one foot behind the other (Stopwatch: Vollo 1809, Brazil) |
| Walking speed | 3 meters distance (Stopwatch: Vollo 1809, Brazil) |
| Blood assays [b] | |
| Total Cholesterol | CHOP-POD Enzymatic Method (Advia 2400, Siemens, Germany) |
| High Density Lipoprotein Cholesterol (HLD) | Elimination/catalase methods (Advia 2400, Siemens, Germany) |
| Low Density Lipoprotein Cholesterol (LDL) | Calculated using the Friedewald equation (Advia 2400, Siemens, Germany) |
| Urea | Enzymatic methods using glutamate dehydrogenase: GLDH (Advia 2400, Siemens, Germany) |
| Creatinine | Colorimetric: Jaffe reaction without deproteination (Advia 2400, Siemens, Germany) |
| Ferritin | Chemiluminometric two-site sandwich immunoassay (Advia Centaur XP, Siemens, Germany) |
| Thyroid Stimulating Hormone (TSH) | Chemiluminescence immunoassay (Advia Centaur XP, Siemens, Germany) |
| Glycated hemoglobin | High performance liquid chromatography: HPLC (Premier Hb9210™ HbA1c Analyzer, Trinity Biotech Plc., Ireland) |
| Vitamin D | Chemiluminescent micro particle immunoassay: CMIA (Architect Rchietec I200SR - Abbott Laboratories, USA |
| Hemogram | Automate (Coulter® LH 750 Hematology Analyzer, Beckman Coulter, Inc, USA) |

a: all study participants; b: sub-sample of study participants
